# Harnessing Virtual Reality: Improving Social Skills in Adults with Autism Spectrum Disorder

**DOI:** 10.3390/jcm13216435

**Published:** 2024-10-27

**Authors:** Andrea Calderone, Angela Militi, Desirèe Latella, Rosaria De Luca, Francesco Corallo, Paolo De Pasquale, Angelo Quartarone, Maria Grazia Maggio, Rocco Salvatore Calabrò

**Affiliations:** 1 Department of Clinical and Experimental Medicine, University of Messina, Piazza Pugliatti 1, 98122 Messina, Italy; 2 Department of Biomedical, Dental Science and Morphological and Functional Images, University of Messina, Piazza Pugliatti 1, 98100 Messina, Italy; angela.militi@unime.it; 3 IRCCS Centro Neurolesi Bonino-Pulejo, S.S. 113 Via Palermo, C.da Casazza, 98124 Messina, Italy; desiree.latella@irccsme.it (D.L.); rosaria.deluca@irccsme.it (R.D.L.); francesco.corallo@irccsme.it (F.C.); paolo.depasquale@irccsme.it (P.D.P.); angelo.quartarone@irccsme.it (A.Q.); mariagrazia.maggio@irccsme.it (M.G.M.); roccos.calabro@irccsme.it (R.S.C.)

**Keywords:** autism spectrum disorder, virtual reality, social skills, neurorehabilitation

## Abstract

**Background and Objectives:** Autism Spectrum Disorder (ASD) involves challenges in social communication and daily functioning. Emerging research highlights that virtual reality (VR) interventions can significantly improve social skills in adults with ASD by providing immersive, controlled practice environments. This systematic review will assess the effectiveness of VR-based interventions for improving social skills in adults with ASD. **Materials and Methods:** Studies were identified from an online search of PubMed, Web of Science, Cochrane Library, and Embase databases without any search time range. This review was registered on Open OSF (*n*) P4SM5. **Results:** Recent studies show that VR interventions significantly enhance job interview skills, social abilities, and practical tasks in adults with ASD, with improvements in confidence, social understanding, and everyday skills. VR has been shown to be user-friendly and effective in providing immersive, adaptable training experiences. **Conclusions:** The review highlights VR’s promising role in improving social skills, job interview abilities, and daily functioning in adults with ASD. It emphasizes the need for broader studies, standardized interventions, and exploration of VR’s integration with other therapies to enhance long-term effectiveness and address comorbidities like anxiety and depression.

## 1. Introduction

Autism spectrum disorder (ASD) is defined by enduring challenges in social communication and interaction, along with limited, repetitive behaviors, interests, or activities [1,2]. The term “spectrum” indicates the diverse range of symptoms in type and severity that individuals may encounter [3,4]. According to data from the Global Burden of Disease (GBD) 2019, ASD represents a significant global health burden. In 2019, there were an estimated 28.3 million prevalent cases of ASD worldwide, with a prevalence rate of 369.4 per 100,000 individuals. Additionally, there were 603,790 new cases of ASD globally (incidence), with an incidence rate of 9.3 per 100,000 individuals. In terms of the disability-adjusted life years (DALYs), the global burden of ASD in 2019 was 4.3 million DALYs, with a rate of 56.3 per 100,000 individuals. This reflects a 39.3% increase in prevalence and a 38.7% increase in DALYs associated with ASD from 1990 to 2019. These statistics highlight the growing impact of ASD on public health, underscoring the need for early diagnosis, targeted prevention, and long-term support strategies. This approach also supports generalization, enabling individuals to apply learned skills to real-world settings, which is a critical aspect of successful rehabilitation. For this reason, VR holds significant promise as an innovative therapeutic modality for improving social functioning in adults with ASD [5,6,7]. Although the specific cause of ASD is still unknown, it is believed to arise from a combination of genetic and environmental influences [8,9]. ASD development is influenced by genetic mutations, toxins, parental age, and birth complications. Abnormalities in the structure and function of the brain also have an influence, demonstrating significant genetic involvement and atypical neural growth [10,11,12]. Regarding social functioning, ASD is identified by issues with social communication and repetitive behaviors, struggles in skill development, as well as commonly co-occurring conditions such as anxiety, depression, epilepsy, and ADHD. Individuals with ASD have difficulty grasping social cues, cultivating relationships, and might be more sensitive to stimuli [13,14,15,16]. Difficulty holding eye contact during conversations may result in confusion and conflicts. Individuals with ASD may find it difficult to understand non-literal language and cultural subtleties, which can impede mutual communication and create obstacles in forming and maintaining friendships and relationships [17,18,19]. Furthermore, they could struggle with grasping societal norms and acceptable behavior, acting in a ways that may be perceived as improper or inconsistent with social standards [20,21]. It can be also difficult to manage daily schedules, prioritize tasks, and complete them on time [22,23,24]. People with ASD frequently encounter challenges with sensory processing, making tasks such as attending school or using public transportation more complex. Their increased auditory and tactile sensitivity could result in feelings of distress and anxiety. Struggling with adjusting to unfamiliar situations or finding solutions to problems can hinder individuals from being independent, necessitating continuous assistance. The involvement in the community is essential for enhancing and promoting these abilities [25,26]. From a rehabilitation perspective, therapeutic measures aim to enhance social, communication, and adaptive skills using customized and research-supported approaches. Behavioral therapy, like Applied Behavior Analysis, centers on rewarding good behaviors and instructing individuals on new skills [27,28]. Speech therapy and occupational therapy can enhance communication skills and improve motor abilities [29]. Furthermore, personalized and tailored educational programs can enhance learning and growth in both school and community environments [30]. Virtual reality (VR) is emerging as a valid tool in the rehabilitation of adults with ASD, particularly in enhancing social functioning and social skills. By simulating real-world environments, VR provides individuals with ASD the opportunity to practice and improve their social interactions in a safe, controlled setting. Research has demonstrated that VR-based interventions can improve skills such as communication, emotional recognition, and perspective-taking, which are often challenging for this population. The immersive nature of VR allows for repeated exposure to social scenarios, facilitating the development of adaptive social behaviors and improving overall social competence [31,32,33]. The VR application offers unparalleled flexibility in the customization and adaptation of therapeutic sessions for individuals with ASD. The immersive nature of VR allows the healthcare team to tailor the virtual environments and scenarios to match the specific needs, abilities, and goals of each patient. This level of personalization is crucial for ASD individuals, who often exhibit unique challenges in social communication and interaction. By adjusting variables such as task difficulty, sensory input, and social cues, VR sessions can be fine-tuned to provide a highly individualized rehabilitation experience that can evolve alongside the patient’s progress, enhancing engagement and outcomes [34,35,36,37]. The interest in using VR therapy to improve social skills in adults with ASD is growing [38,39]. VR offers several advantages in comparison with conventional methods for improving social skills in individuals with ASD. Conventional approaches, such as role-playing or face-to-face interactions, can be limited by the variability of real-life social scenarios and the discomfort many ASD individuals experience in unpredictable environments. In contrast, VR provides a safe, controlled, and repeatable environment where patients can practice social interactions without the anxiety associated with real-world settings. This makes it easier to target specific social deficits, such as initiating conversations or interpreting social cues, and to practice repeatedly in a consistent setting, something not always feasible in real-life social training. Additionally, VR allows for the safe exploration of difficult social situations, such as job interviews or public speaking, without the fear of real-world consequences. This reduces stress and enables patients to build confidence before transferring these skills to real-life scenarios. Furthermore, VR provides immediate feedback, which is critical for learning and improving social behavior. Overall, VR offers a flexible, personalized, and engaging platform for social skills training, enhancing both the efficacy and comfort of rehabilitation for ASD individuals [40,41]. The captivating quality of VR allows users to deeply participate in the training, while the option to replay and personalize scenarios offers chances for ongoing practice and steady enhancement [42]. A major benefit of using VR interventions for social skills training is the capacity to offer instantaneous feedback. Users can receive assistance with their performance, including eye contact, body language, and conversational skills, so they can recognize and fix specific problem areas. This prompt feedback cycle has the potential to speed up learning and strengthen desirable social behaviors [43,44,45,46,47,48,49,50,51]. This systematic review explores the potential of VR-based interventions in enhancing social skills among ASD adults. In determining VR’s efficacy in skill building, the review gives insight into novel approaches that support social inclusion and independence in rehabilitation contexts. Understanding how well VR works in this respect will help bring about future therapeutic practices contributing to better outcomes for adults with ASD.

## 2. Materials and Methods

### 2.1. Search Strategy and Eligibility Criteria

**Figure 1:**
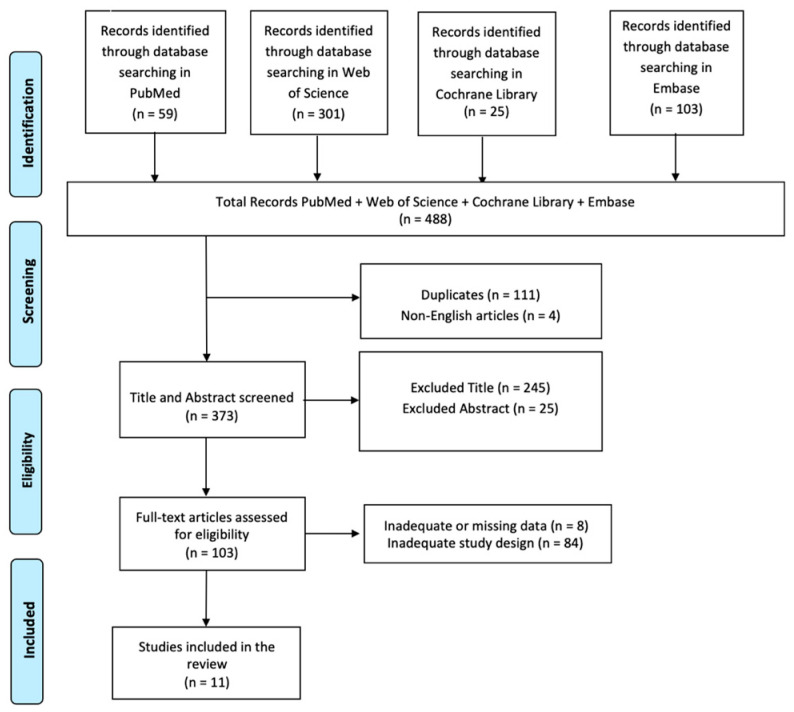
PRISMA 2020 flow diagram of evaluated studies.

A comprehensive literature search was performed using PubMed, Web of Science, Cochrane Library, and Embase databases, employing the keywords: (All Fields: “Autism Spectrum Disorder”) AND (All Fields: “Virtual Reality”) AND (All Fields: “Social Skills”), without any specific search time range. The PRISMA (Preferred Reporting Items for Systematic Reviews and Meta-Analyses) flow diagram was utilized to outline the process (identification, screening, eligibility, and inclusion) for selecting relevant studies, as illustrated in Figure 1. This review has been registered on Open OSF with the identifier: DOI 10.17605/OSF.IO/P4SM5.

All articles were screened based on their titles, abstracts, and full texts by two researchers (AC, DL), who independently performed article collection to reduce the risk of bias (e.g., missing results bias, publication bias, time lag bias, language bias). These researchers read full-text articles deemed eligible for the study, and in case of disagreement regarding inclusion and exclusion criteria, the final decision was made by a third researcher (RCS). Moreover, the agreement between the two reviewers (AC and DL) was assessed using the kappa statistic. The kappa score, with an accepted threshold for substantial agreement set at >0.61, was interpreted to reflect excellent concordance between the reviewers. This criterion ensures a robust evaluation of the inter-rater reliability, emphasizing the achievement of a substantial level of agreement in the data extraction process.

The list of articles was then refined for relevance, reviewed, and summarized, with key topics identified from the summary based on the inclusion/exclusion criteria.

#### 2.1.1. Inclusion Criteria

A study was included if it described or examined the effectiveness of VR-based interventions for improving social skills in adults with ASD. Only articles written in English were considered. Additionally, studies that described or investigated the functional assessment of these patients were included. We included only studies conducted in human populations and published in English that met the following criteria: (i) original or protocol studies of any kind; and (ii) articles that detail the effectiveness of VR-based interventions for improving social skills in adults with ASD.

#### 2.1.2. Exclusion Criteria

A study was excluded if it lacked data or information regarding the effectiveness of VR-based interventions for improving social skills in adults with ASD. Systematic, integrative, or narrative reviews were also excluded; however, their reference lists were reviewed and included when relevant. Additionally, any articles written in languages other than English were excluded.

### 2.2. PICO Evaluation

We applied the PICO model (Population, Intervention, Comparison, Outcome) to create our search terms.

The population targeted in this assessment includes adults diagnosed with ASD. The intervention under scrutiny involves the application of VR-based techniques designed to facilitate improvements in the specified areas. These VR interventions were compared to traditional methods or the absence of any intervention to determine their relative efficacy. The primary outcomes of interest include measurable improvements in social skills, providing a comprehensive understanding of the potential benefits and limitations of VR technology in the treatment and support of adults with ASD. A summary of the materials and methods is visualized in Table 1.

## 3. Results

### 3.1. Quality of Included Studies—Risk of Bias

**Figure 2:**
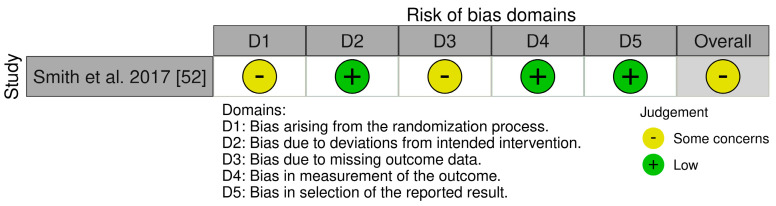
Risk of Bias (RoB2) of included RCT studies.

We assessed the risk of bias using appropriate tools based on the design of the included studies. Of the twelve studies, one was a randomized controlled trial (RCT) [52]. For this one, we used the updated Cochrane Risk of Bias (RoB 2) tool, which covers five domains: (i) bias arising from the randomization process, (ii) bias due to deviations from the intended intervention, (iii) bias due to missing data on the results, (iv) bias in the measurement of the outcome, and (v) bias in the selection of the reported result (Figure 2) [53].

**Figure 3:**
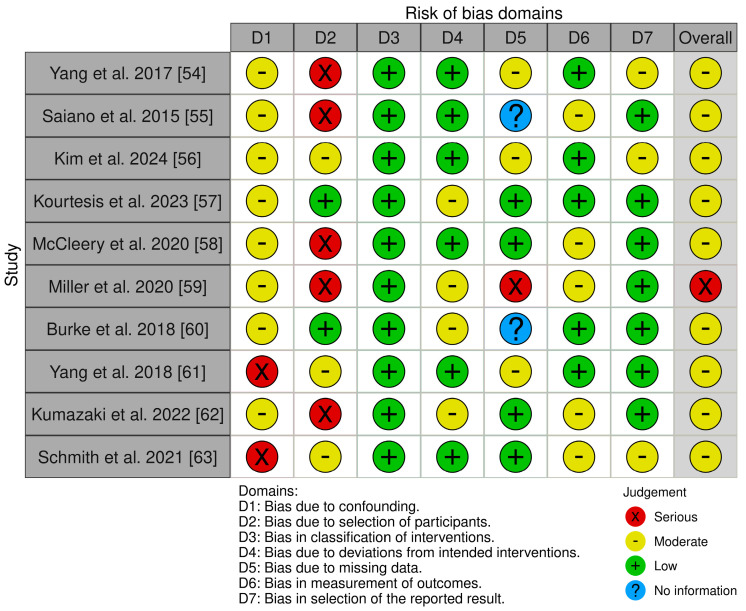
Cochrane Risk of Bias in Non-randomized Studies of Interventions (ROBINS-I).

Our assessment identified a moderate risk of bias and modest methodologies. We found some concerns due to bias arising from the randomization process due to lack of some information (D1) and bias due to missing outcome data (D3). For the ten non-randomized studies—one preliminary pilot study [54], one clinical trial [55], two within-subjects study [56,57], one systematic examination study [58], one pilot study [59], three interventional studies [60,61,62], and one multi-phase usage study [63]—we applied the ROBINS-I tool. It assesses bias in seven areas: (i) bias due to confounding, (ii) bias in participant selection, (iii) bias in classification of interventions, (iv) bias due to deviations from intended interventions, (v) bias due to missing data, (vi) bias in outcome measurement, and (vii) bias in selection of the reported outcome (see Figure 3) [64].

The ROBINS-I ratings of the ten studies indicate a generally moderate methodological quality, although each study has notable areas of concern. Yang et al. [54], Saiano et al. [55], McCleery et al. [58], Miller et al. [59], and Kumazaki et al. [62] demonstrate a moderate overall risk of bias (except one [59]), reflecting some moderate issues in D1 (bias due to confounding) and serious risk in D2 (bias in the selection of participants). However, Yang et al. [54], Saiano et al. [55], Kim et al. [56], McCleery et al. [58], Yang et al. [61], and Schmith et al. [63] display a low risk of bias in D3 (classification of interventions) and D4 (deviations from intended interventions). Furthermore, all the studies selected show a low risk of bias in D3. No information was detected in the area of missing data (D5) in the papers by Burke et al. [60] and Saiano et al. [55]. All articles presented a low risk of bias in the selection of the reported results, except for the studies by Yang et al. [54], Kim et al. [56], and Schmith et al., which instead showed a moderate risk. Miller et al. [59] display a serious overall risk of bias thanks to serious difficulties in the D2 and D5 domains. Kumazaki et al. [62], Burke et al. [60], Miller et al. [59] and Kourtesis et al. [57] presented a moderate risk of bias in D4, while Saiano et al. [55], McCleery et al. [58], Miller et al. [59], Kumazaki et al. [62], and Schmith et al. [63] also showed a moderate risk in the outcomes measurement (D6). In general, although many studies indicate a moderate bias risk in various areas, it is essential to thoroughly assess elements like confounding factors, outcome measurement, and participant selection to guarantee the trustworthiness and accuracy of the study findings.

### 3.2. Synthesis of Evidence

In total, 488 articles were found: 111 articles were removed due to duplication after screening; four articles were excluded because they were not published in English; a total of 270 articles were excluded based on title and abstract screening. Finally, 92 articles were removed based on screening for inadequate and untraceable study designs (Figure 1). Eleven research articles met the inclusion criteria and were therefore included in the review. These studies are summarized in Table 2.

The studies discussed in this review explored the potential of VR-based interventions for improving social skills, communication, and adaptive behaviors in adults with ASD. Four articles analyzed the potential of VR in job interview training and social skills development [52,54,55,56], while seven papers examined the role of VR in enhancing practical/social skills and user experience [57,58,59,60,61,62,63].

### 3.3. Potential of VR in Job Interview Training, Communication, and Social Skills Development

Researchers are more and more examining how VR can help people with ASD in different areas, like job interview practice, social skills development, and everyday tasks. A first randomized controlled trial with 79 participants with severe mental illness or ASD was conducted to evaluate the effects of VR job interview training on interviewing skills and job offers. Findings indicated that the more virtual interviews completed, the higher the improvement in post-test interviewing skills, leading to a greater probability of getting a job offer within six months [52]. A preliminary examination studied neuroimaging biomarkers to forecast the efficacy of Virtual Reality-Social Cognition Training (VR-SCT) in 17 young adults diagnosed with high-functioning ASD. By utilizing a biological motion neuroimaging task, scientists discovered that the brain activations before treatment in neural circuits associated with language understanding and socio-emotional processing could forecast the enhancement in emotion recognition following VR-SCT [54]. A clinical trial assessed the effectiveness of virtual environments in teaching safety skills, particularly pedestrian skills, to seven adults with ASD. Participants used a motion capture device to explore a digital city during ten 45-min sessions held weekly. Six participants finished the procedure, displaying notable enhancements in navigation abilities without improvement in decreasing street crossing mistakes. Even though there were no noticeable differences in test questionnaire mistakes, parents and caregivers reported significant enhancements in actual street-crossing abilities, suggesting a successful transfer of skills [55]. Kim et al. assessed how WorkplaceVR, a VR system, helped 14 young adults with autism manage work-related social situations by examining their behavioral and physiological reactions. Through a study that included both within-subject deployment and mixed methods, the findings showed a notable boost in participants*’* perceived self-efficacy following their utilization of the system. The thematic analysis indicated an improved understanding of anxiety triggers and behaviors, leading participants to reflect on actual experiences and create strategies for managing anxiety. The VR technology encouraged participants to advocate for themselves more and boosted their confidence in workplace social interactions [56].

### 3.4. VR’s Role in Enhancing Practical and Social Skills and User Experience for Individuals with ASD

VR is becoming an effective tool for improving skills in individuals with ASD, with studies showing its potential in areas like job interview practice, social skills development, and everyday life abilities. McCleery et al. investigated how safe, possible, and easy to use an immersive VR training program was for 60 people with ASD aged 12 and older who could can speak fluently. The participants were split into two groups of 30 people each and received either one or three 45-min VR sessions with a portable wireless headset while being observed for any negative effects. The results verified that immersive VR is secure and extremely user-friendly for this group. Participants expressed strong enjoyment and a willingness to participate in more VR sessions, suggesting VR*’*s effectiveness in teaching practical life skills to those with ASD [58]. In another paper, 25 ASD participants received social skills training in VR, which showed a high level of acceptability, usability, and enjoyment. Participants were given cognitive tests and exposed to different social scenarios in VR. Results showed that self-assessment scores were highly correlated with executive function and performance in social scenarios, with the variable of planning ability acting as the strongest predictor of success. Immersive VR was an effective intervention for teaching socially relevant skills, using individualized strategies without errors [57]. Another pilot study examined the utilization of VR for instructing air travel abilities to seven autistic young adults, employing a modified virtual airport setting. Participants utilized a 5-min VR experience weekly for three weeks, employing an iPhone X (Apple Inc. Cupertino, CA, USA) and Google Cardboard (Google LLC., Mountain View, CA, USA). The research evaluated enhancements in focus, language skills, understanding of activities, and clinical observations related to interaction with technology. The findings indicated notable improvements in focus, word recognition, and task understanding. Clinical assessments verified that the VR was well-received and showed promise as a beneficial intervention method [59]. Burke et al. examined how the Virtual Interactive Training Agent (ViTA) system helps enhance job interviewing skills for 32 individuals with autism and developmental disabilities. Researchers found a notable rise in Marino Interview Assessment Scale scores using a linear mixed model to analyze data from the initial ViTA session to the ultimate face-to-face interview. Participants showed improved abilities in recognizing strengths, self-marketing, self-assertion, and addressing situational and behavioral inquiries, indicating that the ViTA system effectively aids in job interview readiness through role-playing experiential training [60]. Yang et al. analyzed alterations in neural and behavioral responses in young adults with high-functioning ASD after participating in VR-Social Cognition Training for five weeks (10 h total). By utilizing neuroimaging during a biological/social versus scrambled/nonsocial motion task, the research discovered three main results: heightened activity in the right posterior superior temporal sulcus was connected to advancements in theory-of-mind, diminished activity in the left inferior frontal gyrus was tied to better emotion recognition, and decreased activity in the left superior parietal lobule was related to decreased attention to nonsocial stimuli [61]. Another study created a VR intervention as a proof-of-concept to improve public transportation abilities in adults with autism, utilizing 360-degree video and VR headsets. The intervention was created using complexity and generalization theories, incorporating a staged method and slowly reducing technological and pedagogical assistance. This study evaluated the efficiency and appeal of the intervention by involving four expert testers and five participant testers with autism throughout several stages of usage. Results disclosed that learners had a generally positive experience, and the intervention was found to be both feasible and appropriate for the specific group of people [63]. A final trial evaluated the efficiency of an online job interview training program for individuals with ASD, using virtual robots in group sessions. The project included 15 individuals who participated in 25 sessions, alternating between the positions of interviewee, interviewer, and evaluator. Assessments were carried out before and after the training through simulated interviews with an expert human interviewer. The findings indicated that participating in Game of Thrones significantly boosted individuals*’* self-assurance, drive, and performance in interviews, while also increasing their empathy towards others. Significant progress was seen in both verbal and non-verbal skills and in understanding the perspectives of interviewers [62]. VR proves effective in supporting persons with ASD in acquiring skills in job interviews, social interactions, and instrumental activities of daily living. Evidence may state that VR treatment would boost skills in job interviews, social understanding, and adaptive behavior, fostering greater confidence and efficiency.

## 4. Discussion

This systematic review explores the potential of VR-based interventions for improving social skills, communication, and adaptive behaviors in adults with ASD. The studies reviewed unveiled that VR interventions could be successful in improving different abilities in individuals with ASD. Indeed, it was shown that VR can greatly enhance abilities in job interviews, social interactions, and daily life tasks [52]. For example, utilizing VR for job interview practice has been shown to improve interviewing skills after the test and boost the chances of getting job offers. Neuroimaging markers can forecast enhancements in emotion identification after undergoing VR-Social Cognition Training. Pedestrian safety training has illustrated that VR environments are also successful in teaching safety and navigation skills. Furthermore, VR platforms such as WorkplaceVR and ViTA have effectively assisted people with ASD in navigating social interactions at work and enhancing their preparedness for job interviews. Research demonstrates that VR training programs are deemed safe, easy to use, and enjoyable, leading participants to outline eagerness for additional sessions. Moreover, VR treatments have shown success in educating on interpersonal abilities and enhancing skills in navigating public transportation. In general, VR technology is demonstrated to be a valuable tool in treating autism, offering tailored, realistic training settings that improve practical and social abilities, increase self-esteem, and enhance user satisfaction [54,55,56,57,58,59,60,61,62,63]. This review provides an in-depth understanding of how virtual environments can be individually tailored to support some common deficits among ASD, such as job interview preparation, pedestrian safety, and workplace interactions [65,66]. What is particularly novel here is that VR consistently demonstrates improvement not only in these practical skills, but also in increased self-efficacy, emotion recognition, and social cognition, especially when neuroimaging data supports these findings, offering insight into associated changes in brain activity [67,68,69,70].

Current literature has long suggested that immersive, simulated environments provide the individual with ASD with the ability to practice social interactions and adaptive behaviors under low-pressure conditions. The studies included in our review confirm this important issue, but the integration of neuroimaging biomarkers is relatively new. Predicting improvement in emotion recognition based on neural activity in regions involved in social and linguistic processing adds an objective and measurable component to VR interventions [71,72,73]. The included findings also extend existing literature to demonstrate not only that VR is effective in skill acquisition, but also that it is highly accepted by users [69]. Research highlights the importance of user experience, showing that individuals with ASD find VR enjoyable, safe, and highly usable [69]. This fills an important gap compared to previous research, which has focused more on technical effectiveness but paid little attention to participant engagement and motivation [74]. This is further reinforced by the fact that skills learned within VR were successfully transferred to real life, such as crossing the street more safely and better prepared for job interviews, suggesting that functional gains could be realized from VR interventions [75,76]. With that in mind, more research is still needed in this area. While some studies report promising results [65,66], others suggest that transferring skills from virtual to real-world settings is not always straightforward [77,78].

From a clinical perspective, data from this systematic review may support the integration of VR into neurorehabilitation programs in ASD, enabling personalized, immersive environments that can be easily manipulated in real-time to accommodate the participant**’**s learning pace and needs—a huge advantage over traditional therapeutic methods [79,80]. The importance of incorporating errorless learning strategies when working in a VR environment is also highlighted [81]. Participants can develop certain skills without any apprehension of making a mistake, which is a major concern for most individuals with ASD [82,83]. A limitation that impacts these studies, as well as the broader literature, is the focus on high-functioning individuals with ASD [83]. In most of these investigations, participants included young adults demonstrating higher-functioning forms of autism, which raises open questions about the generalizability of these interventions to those individuals with more severe cognitive or social disabilities [84]. Accordingly, the results of this study are promising but do not reveal the full potential of VR-based intervention for individuals across the autism spectrum. Further research in this line should investigate how VR can be adapted to meet the needs of individuals with more significant needs through the addition of other assistive technologies or personalized intervention methods [85].

The review shows significant strengths by providing an in-depth evaluation of VR interventions aimed at improving social skills in adults with ASD. Its comprehensive incorporation of various research methods and outcomes is a major advantage, providing a comprehensive perspective on the effectiveness of VR. By including results from randomized clinical trials, neuroimaging studies, and clinical trials, as well as their risk of bias analysis, the review provides a comprehensive evaluation of the effects of VR on skills for job interviews, social interactions, and daily activities in adults with ASD [69,71]. This comprehensive strategy aids in grasping the wide range of uses for VR interventions and strengthens the technology**’**s ability to improve social skills and independence [68,74]. Another advantage lies in the review**’**s focus on the satisfaction of users and the safety of VR interventions [70,71]. The studies demonstrate that VR training is widely accepted, easy to use, and interesting, which is important for continued involvement and success [69,75]. The review also highlights how VR environments can be customized to cater to individual requirements and sensitivities, providing a personalized method for improving skills [73,76].

Nevertheless, there are some restrictions in the review. The diversity in research designs, VR interventions, and outcome measures creates difficulty in reaching clear conclusions or making direct comparisons between studies. Moreover, only eleven articles met the inclusion criteria and the absence of standardization could impact the general applicability of the results. Several of the studies analyzed in the review have limited follow-up periods and small sample sizes, which could affect the credibility and long-term relevance of the findings. Furthermore, the review mainly focuses on studies published in English, which could overlook important findings from research in other languages. Additionally, although the review highlights the possibilities of VR interventions, it does not thoroughly discuss real-world obstacles to integrating VR technology. Topics like the price of VR gear, availability, and variations in VR encounters are not fully investigated.

### 4.1. Clinical Applications

The clinical applications of VR technologies for adults with ASD are broad and promising, particularly in improving social and communication skills. VR provides safe and controlled environments where patients can practice complex social situations, such as job interviews or everyday interactions, reducing anxiety associated with direct social engagement. This technology can be integrated into clinical practice through supervised training sessions conducted by trained therapists, who can adapt virtual scenarios to the specific needs of the patient. Furthermore, the ability to personalize interventions offers greater flexibility compared to traditional methods, facilitating the implementation of targeted therapeutic programs that address different levels of ASD severity. Importantly, integrating patient feedback into the design of VR experiences ensures that interventions remain relevant and effective.

The integration of VR into rehabilitation could also promote greater autonomy, as skills learned in virtual environments can be transferred and generalized to real-life situations, ultimately improving the quality of life of adults with ASD. However, it is crucial to consider challenges such as accessibility and the need for specialized training for practitioners. Future research should explore these areas further to optimize the effectiveness and implementation of VR technologies in clinical settings.

### 4.2. Limitations of the Study

Despite the promising findings of this review on the use of VR in ASD, there are several limitations that need to be addressed. A significant issue is the variability in methodological rigor among the studies included in this review. Not all studies were randomized controlled trials, which limits the generalizability of the findings. Additionally, the review did not include a meta-analysis, which could have provided a more robust statistical understanding of the overall effectiveness of VR interventions for this population. Key databases, such as PsycINFO and Scopus, were not included, potentially limiting the scope of the literature reviewed and missing relevant studies that could have contributed to a more comprehensive understanding of the topic.

Another limitation is the relatively small sample sizes in many of the studies, which makes it difficult to draw firm conclusions about long-term outcomes. Future research should aim to standardize protocols and include larger randomized samples to improve the reliability of the findings and provide a stronger evidence base for clinical applications.

## 5. Conclusions

The systematic review indicates that VR interventions could provide benefits for adults with ASD in terms of social skills, communication, and adaptive behaviors. Social functioning in adults was shown to improve through VR, including job interview skills, social interaction, and activities of daily living. It provides a structured environment in which people can develop their skills and build confidence in real-life interactions. To confirm these findings and maximize effectiveness, larger research samples are required to ensure generalizability across a variety of populations and settings. Standardizing VR interventions will ensure greater consistency and effectiveness. Other areas of research that should be explored include cost-effectiveness, individualization of VR experiences to meet specific needs, combination with other therapies, and addressing comorbidities such as anxiety and depression. Finally, studies should investigate the long-term effectiveness of VR treatments and analyze how to minimize sensory overload and enhance relaxation during virtual exposure.

## Figures and Tables

**Table 1:** Summary materials and methods.

Population	Intervention	Comparison	Outcome	Search Strategy	Inclusion Criteria	Exclusion Criteria	Limitations of the Review
Adults diagnosed with autism spectrum disorder	Virtual Reality based techniques for improving social skills	Traditional methods or absence of any intervention	Measurable improvements in social skills	Databases: PubMed, Embase, Cochrane Library, Web of Science. Search String: (Autism Spectrum Disorder OR ASD) AND (adults) AND (Virtual Reality OR VR) AND (social skills) AND (intervention OR therapy OR treatment) Search time range: none.	Studies describing or examining the effectiveness of virtual reality-based interventions for improving social skills in adults with autism spectrum disorder. Studies published in English. Studies focusing on human populations. Original research or protocol studies of any type. Articles detailing functional assessment or improvements in social skills.	Studies lacking data on the effectiveness of virtual reality-based interventions for improving social skills in adults with autism spectrum disorder. Systematic, integrative, or narrative reviews (although their references may be considered if relevant). Articles published in languages other than English.	Variability in methodological rigor among the included studies, with not all studies being randomized controlled trials (RCTs), limiting the generalizability of the findings. The review did not include a meta-analysis, which could have provided more robust statistical insights into the effectiveness of VR interventions. Key databases, such as PsycINFO and Scopus, were not included, potentially limiting the scope and comprehensiveness of the literature review. Many studies had small sample sizes, making it difficult to draw firm conclusions about long-term outcomes.

**Table 2:** Summary of studies included in the research.

Author	Aim	Study Design/Intervention	Treatment Period	Sample Size	Sample Characteristics	Outcomes Measures	Main Findings
Smith et al., 2017 [52]	To evaluate whether improved interviewing skills, gained through VR-JIT, mediated the relationship between completing virtual interviews and receiving job offers within six months.	Randomized Controlled Trial.	From January 2012–May 2014.	A total of 79 trainees with mental disorders or ASD who participated in the VR-JIT program.	Age: The participants had an average age of 43.1 ± 14.8 years. Gender: There were a total of 56 males, making up 71% of the group. Clinical Scores: Correlation with Post-test Interview Skills: r = 0.82, *p* < 0.001	Pre- and post-test mock interviews.	The study found that completing more virtual interviews predicted improved interviewing skills, which, in turn, predicted a higher likelihood of receiving a job offer. Both relationships were statistically significant.
Yang et al., 2017 [54]	To identify pretreatment biomarkers using neuroimaging that could predict the response to VR-SCT in young adults with high-functioning ASD.	Uncontrolled Experimental Studies.	17 young adults with high-functioning ASD.	A total od 17 young adults with high-functioning ASD.	Age: 22.50 years Gender: 2 females, 15 males. Clinical Scores: The research indicated a substantial enhancement in socio-emotional processing abilities after VR-SCT intervention, with notable progress from before treatment (M = 7.63, SD = 3.42) to after treatment (M = 9.63, SD = 3.78), supported by a Cohen’s drm of 0.55 (*p* = 0.03).	MRI.	Neural predictors of changes in emotion recognition after VR-SCT were identified. Key predictors included brain activations related to language comprehension, socio-emotional experience, and emotional regulation. The study demonstrated that these predictors could forecast individual responses to VR-SCT.
Saiano et al., 2015 [55]	To evaluate the effectiveness of an integrated approach using virtual environments and natural interfaces to teach pedestrian safety skills (e.g., street crossing, following road signs) to adults with ASD.	Uncontrolled experimental studies	Ten sessions, with each session being 45 min long, conducted weekly.	Seven adults with ASD.	Age: 29 ± 10 years (range: 19–44) Gender: All males. Clinical Scores: The error rate for ‘crossing with red/yellow light’ showed a notable decrease (*p* = 0.0313; Wilcoxon’s sign test) from pre-treatment (T0) to post-treatment (T1) evaluations.	A test questionnaire was administered before and after treatment to assess understanding of the practiced skills, and a separate questionnaire for parents/legal guardians and caregivers to evaluate real-life skill transfer.	Participants showed better navigation skills in the virtual environment but did not show a significant decrease in errors during street crossing, as found in the study. Despite this, parents and caregivers reported significant improvements in street crossing skills in real-life situations, indicating successful skill transfer.
Kim et al., 2024 [56]	To create and assess WorkplaceVR, a virtual reality system that aims to boost the self-confidence of individuals with autism in social workplace situations by combining simulated experiences with data visualizations of their responses.	Uncontrolled experimental studies	Not Specificated.	Fourteen young adults with autism participated in the study.	Age: 16 to 34 years. Gender: 2 females, 12 males. Clinical Scores: Not Specificated.	PSES-VR	The VR system significantly improved participants’ self-efficacy, with a notable increase in perceived self-efficacy. Thematic analysis revealed that participants gained better self-awareness about their anxiety triggers and behaviors, which encouraged them to reflect on their experiences and develop self-advocacy strategies.
McCleery et al., 2020 [58]	To evaluate the safety, feasibility, and usability of an immersive VR training program for adolescents and adults with ASD, focusing on its potential to help develop practical life skills.	Uncontrolled experimental studies	The intervention involved either one or three VR sessions, each lasting for 45 min.	Sixty verbally fluent individuals with ASD participated in the study.	Age: 12–38 years old Gender: males and females (not specificated). Clinical Scores: The System Usability Scale adapted for verbally fluent adolescents and adults with ASD (SUS-ASD). For Cycle A, the SUS-ASD score averaged 83.58% (SD: 12.49%; range: 52.5–100%), indicating good usability. For Cycle B, the SUS-ASD score averaged 87% (SD: 7.89%; range: 67.5–100%), suggesting strong usability over multiple sessions.	Recordings and semi-structured qualitative interview.	The study found that immersive VR is safe and feasible for use in verbally fluent adolescents and adults with ASD. The VR sessions were well-received, with high usability and enjoyment reported by participants.
Kourtesis et al., 2023 [57]	To evaluate the acceptability, usability, and user experience of a VR social skills training program designed for individuals with ASD. It also sought to understand the relationship between performance in VR social scenarios and various cognitive functions.	Uncontrolled experimental studies	Not Specificated.	A total of 25 participants with ASD.	Age: from 19 to 52 years. Gender: 6 females, 19 males. Clinical Scores: at the task completion score most participants showed a ceiling effect (high scores with little variance).	Tower of London, Stroop Test, Digit Recall, Questionnaire, UEQ, SUS, CSQ-VR.	The VR social skills training was highly accepted by participants, and it was found to be usable with a positive user experience. Performance in social scenarios, self-reports, and executive functions were significantly correlated. In particular, the ability to plan was a significant factor in predicting performance in social situations and the usability of VR systems.
Miller et al., 2020 [59]	To evaluate the feasibility and effectiveness of using VR to teach air travel skills to autistic young adults. The objective was to determine if VR can improve attentiveness, language functions, and activity comprehension in this population.	Uncontrolled experimental studies	The VR-ATT program consisted of a single 20-min session each week over a period of three weeks.	Seven participants on the autism spectrum.	Age: The average age was 18.28 years (ranging from 10 to 22 years), including six individuals aged 16 to 22 years and one individual aged 10 years. Gender: 1 female, 6 males. Clinical Scores: (Air Travel Skill Acquisition): Test scores for comprehension improved by 10% from the initial session to the final session. Two participants had lower scores in the final test than in the first, whereas four participants demonstrated enhancement.	Comprehension tests.	The study found improvements in attentiveness, specific language functions like vocabulary labeling, and activity comprehension among most participants. Clinical observations indicated that the VR technology was acceptable and engaging for the participants, suggesting its potential for delivering effective interventions.
Burke et al., 2018 [60]	To evaluate whether using a system could enhance job interviewing skills in individuals with autism and developmental disabilities.	Uncontrolled experimental studies	The treatment period consisted of sessions with the ViTA system, culminating in a final face-to-face interview.	A total of 32 participants with autism and developmental disabilities.	Age: 19 to 31 years old (Mean = 23, SD = 3.12). Gender: Majority males (*n* = 25, 78.13%). Clinical Scores: There was a significant 0.58 unit increase in the average MIAS score (SE = 0.12, t(118) = 5.04, *p* < 0.0001) from the initial ViTA session to the last in-person interview.	MIAS.	The research revealed a significant improvement in participants’ MIAS scores from the initial ViTA session to the last in-person interview. Participants showed enhanced skills in identifying strengths, self-promotion, self-advocacy, and responding to various interview questions.
Yang et al., 2018 [61]	To investigate neural and behavioral changes in young adults with high-functioning ASD following a VR-SCT intervention, providing evidence on the neural mechanisms underlying behavioral improvements.	Uncontrolled experimental studies	Five weeks, with a total of 10 h of VR-SCT intervention.	A total of 17 young adults with high functioning of ASD.	Age: M = 22.50 years, SD = 3.89 Gender: 2 females, 15 males. Clinical Scores: The main clinical measure focused on emotion recognition, showing a noteworthy enhancement from before treatment (M = 11.41, SD = 4.42) to after treatment (M = 12.94, SD = 3.51), Δ = 1.53, t(16) = 2.32, *p* = 0.03, Hedges’s g_av = 0.38.	Advanced Clinical Solutions for WAIS-IV and WMS-IV Social Perception Subtest.	Three significant brain-behavior changes were identified: increased activation in the right posterior superior temporal sulcus correlated with gains in theory-of-mind, highlighting improved socio-cognitive processing; Less activation in the left inferior frontal gyrus was associated with improved emotion identification.
Schmith et al., 2021 [63]	To develop and evaluate a proof-of-concept adaptive skills intervention using VR for adults on the autism spectrum, focusing on promoting the safe and appropriate use of public transportation.	Uncontrolled experimental studies	Summer 2018.	Nine participants: four expert testers and five participant testers with autism.	Age: The average age of the participants was 26.2 years, ranging from 22 to 34 years old. Gender: Not specificated. Clinical Scores: SUS score for Google Cardboard: 79.38; SUS score for Google Daydream: 84.38; Average SUS score across all participants: 79.58 (SD = 0.99); Average SUS score for expert reviewers: 81.88	Interview post exercises with VR.	The results indicate a positive learner experience with feasible and relevant intervention for adults on the autism spectrum, promoting skill acquisition and generalization effectively.
Kumazaki et al. 2022 [62]	To develop and evaluate the effectiveness of a GOT to help individuals with ASD acquire online job interview skills.	Exploratory pilot study	A total of 25 sessions for each participant.	A total of 15 individuals with ASD.	Age: 21.1 years (SD = 1.8). Gender: Male. Clinical Scores: The primary discovery of the research is demonstrated by the self-confidence score, which showed a significant improvement after the second simulated online interview in comparison to the first (3.27 vs. 2.27, *p* = 0.002).	Mock online job interviews.	The GOT training program resulted in notable enhancements in the self-assurance, drive, empathy, communication skills, and overall interview ratings of the participants.

Legend: Autism Spectrum Disorder (ASD), Virtual Reality-Social Cognition Training (VR-SCT), Magnetic Resonance Imaging (MRI), Virtual-Reality job interview training (VR-JIT), Virtual Reality (VR), Perceived Self-Efficacy for VR Social Skill Training Scale (PSES-VR), User Experience Questionnaire (UEQ), System Usability Scale (SUS), Cybersickness in Virtual Reality Questionnaire (CSQ-VR), virtual reality air travel training (VRATT), Virtual Interactive Training Agent (ViTA), Marino Interview Assessment Scale (MIAS), Virtual Reality-Social Cognition Training (VR-SCT), System Usability Scale (SUS), group-based online job interview training program using a virtual robot (GOT).
